# Winning a CHSH Game without Entangled Particles in a Finite Number of Biased Rounds: How Much Luck Is Needed?

**DOI:** 10.3390/e25050824

**Published:** 2023-05-21

**Authors:** Christoph Gallus, Pawel Blasiak, Emmanuel M. Pothos

**Affiliations:** 1 THM Business School, Technische Hochschule Mittelhessen, D-35390 Gießen, Germany; 2 Institute for Quantum Studies, Chapman University, Orange, CA 92866, USA; blasiak@chapman.edu; 3 Institute of Nuclear Physics, Polish Academy of Sciences, PL-31342 Kraków, Poland; 4 Psychology Department, City, University of London, London EC1V 0HB, UK; emmanuel.pothos.1@city.ac.uk

**Keywords:** CHSH games, Bell inequalities, secure quantum communication, quantum information

## Abstract

Quantum games, such as the CHSH game, are used to illustrate the puzzle and power of entanglement. These games are played over many rounds and in each round, the participants, Alice and Bob, each receive a question bit to which they each have to give an answer bit, without being able to communicate during the game. When all possible classical answering strategies are analyzed, it is found that Alice and Bob cannot win more than 75% of the rounds. A higher percentage of wins arguably requires an exploitable bias in the random generation of the question bits or access to “non-local“ resources, such as entangled pairs of particles. However, in an actual game, the number of rounds has to be finite and question regimes may come up with unequal likelihood, so there is always a possibility that Alice and Bob win by pure luck. This statistical possibility has to be transparently analyzed for practical applications such as the detection of eavesdropping in quantum communication. Similarly, when Bell tests are used in macroscopic situations to investigate the connection strength between system components and the validity of proposed causal models, the available data are limited and the possible combinations of question bits (measurement settings) may not be controlled to occur with equal likelihood. In the present work, we give a fully self-contained proof for a bound on the probability to win a CHSH game by pure luck without making the usual assumption of only small biases in the random number generators. We also show bounds for the case of unequal probabilities based on results from McDiarmid and Combes and numerically illustrate certain exploitable biases.

## 1. Introduction

John Bell’s seminal ideas [1,2,3,4,5,6] have allowed a precise and quantifiable illustration that quantum mechanical entanglement enables experimental outcomes which cannot be classically generated. Such presentations of the quantum edge can come in different forms, for example, the Mermin–Peres magic square game, the Greenberger–Horne–Zeilinger (GHZ) game or the Clauser–Horne–Shimony–Holt (CHSH) game. These also come with different story lines, such as hypothetical TV quizzes, polls or trials [7,8,9,10,11,12,13]. The goals of these presentations are also different. They may concern teaching quantum information theory or illustrating the power of entanglement, for example, for uses in quantum cryptography or quantum computation, as well as in discussions of foundational questions of locality and free choice. These games provide a good and accessible illustration of the philosophical puzzle and the practical power of entanglement while only using terms from macroscopic experience.

When using games as illustrations, it is important to also treat the effect of purely random fluctuations when only a finite number of rounds are played. Sweeping the issue of a finite number of rounds *N* under the rug takes away a lot from the usefulness of such presentations. It is equally important to consider imperfect probability distributions. Our contribution lies in a rigorous and accessible treatment of CHSH games and the chances of winning in a finite number of rounds with the additional difficulty that measurement settings cannot be ensured to occur with equal probabilities. This topic is not only relevant for illustrative games, but also has practical implications: in the implementation of quantum devices for the use of quantum technologies, imperfect joint probability distributions may arise, such as an undesired drifting of states or crosstalk between different qubits [14]. Detection and the possible correction of such issues is important in various applications such as the detection of eavesdropping in quantum cryptography [15,16], the fidelity of quantum random number generators [17] or the general witnessing of entanglement [18].

In the present work, we only discuss CHSH games, for which all questions are generated freely and independently. We assume a finite number of game rounds and we do not insist that all measurement settings occur with equal probability. We made this choice of topic for three reasons. First, the CHSH form can be tested well with current experimental technology. Second, the CHSH form of the Bell inequalities can be symmetrically formulated with expectation values and is so open for applications to macroscopic systems outside physics [19,20]. In such applications, the number of rounds is naturally finite and there is no a priori reason for which measurement settings would occur with equal likelihood. The number of rounds can be quite low so that random fluctuations play a greater role than in photon-based physical experiments. This is particularly problematic when historical data are considered where the causal regime at work is suspected to have changed over time. Lastly, CHSH games are rigid, so that strategies with maximum success probability are isomorphic [21,22,23], allowing a generic way of treatment.

Statistical effects from random fluctuations over a finite number of rounds have of course previously been discussed in the literature. The main focus has been the analysis of Bell experiments to disprove local hidden variable models while not allowing loopholes. Statistical confidence in the conclusion from Bell experiments is usually derived by observing that violations of Bell inequalities happen with a certain number of standard deviations, but this line of argument typically requires the assumption of a Gaussian error distribution and the independence of the game rounds, which makes it problematic [24]. We therefore do not follow this line of argument here, but start from early work from Gill [25,26] on this topic, which was later extended in [27]. Such later work yielded sharper bounds, but references, usually without proof, very technical mathematical tools (based on the concentration inequalities from Hoeffding–Azuma, McDiarmid, and others) with the goal of aiding specialists in designing and evaluating Bell experiments. Putting such technical work to use, even by specialists, may still have its pitfalls, as pointed out in [28]. Here, we strive for an accessible presentation that yields interesting and illustrative bounds for situations with unequal probabilities for the measurement settings, when games are played over a finite number of rounds. In contrast to [27], we are interested in random number generator biases that can be very large and we want to avoid linear programming or martingale methods.

The present paper is organized as follows: in Section 2, we present two versions of the CHSH game, one in which the players have to achieve a high percentage of winning rounds and one in which the players have to achieve a high *S*-value, and comment on the equivalence of the versions. In Section 3.1, we proved an extension of Gill’s result [26] for the case of unequal probabilities. The proof is self-contained and only requires an elementary knowledge of probability. We continue in Section 3.2 by proving a bound for unequal probabilities based on McDiarmid Inequality. We also simulate certain game strategies in Section 4 and illustrate some exploitable biases. The last section concludes with a short summary.

## 2. The CHSH Game

Using the notion of the CHSH game, the additional resource that entangled particles may bring can be easily illustrated without the need to understand quantum mechanics. To this end, imagine a game, which is played over many rounds, in which Alice and Bob work as partners trying to win. We assume that they can jointly receive a prize, which is awarded to them by a Verifier based on a clear statistical pre-agreed rule after all game rounds have been played. Alice and Bob may discuss various pre-arranged strategies before the game commences, but they are isolated and not allowed to communicate once the game has started. The game shall be played over *N* rounds and in each round, Alice and Bob will each receive one of two possible inputs (called *questions*) to which they have to give one of two possible outputs (called *answers*). Once the information is brought together, the complete list of questions and answers can be used by an independent Verifier to generate a statistic from which he or she will determine whether Alice and Bob deserve the prize. We will present two versions of the CHSH game, one in which Alice and Bob have the task of winning in more than 75% of the rounds, and one in which they have the task of bringing a statistical quantity (the so-called *S-value*, computed from four expectation values that can be formed from the game statistic) above the value of 2. We will then show how these two versions relate to each other.

### 2.1. The Game with the Goal of Achieving a High Percentage of Winning Rounds

In this version of the game, the task of Alice and Bob is presented as receiving local input bits *x* and *y* (*questions*) from which they have to produce local output bits *a* and *b* (*answers*), such that

(1) $$\begin{array}{c} x\cdot y=a⊕b \end{array}$$

holds. Whenever that happens, Alice and Bob have won the round, otherwise, they have lost the round. In this equation, the symbol ⊕ denotes an addition modulo 2 and · denotes ordinary multiplication. All variables x,y,a,b have to be in the set {0;1}.

This means that, in each round, there is a specific question regime, i.e., a pair (x,y), which we write in shorthand as xy:=(x,y). The key point is that the question regime in a given round xy∈{00,01,10,11} is not fully known to Alice and Bob during the game, and it can only be determined when the data are brought together and analyzed jointly. During the game, Alice has to determine *a* based on *x* without knowing *y*, and Bob has to determine *b* based on *y* without knowing *x*.

Therefore, the set of all possible *elementary strategies*, i.e., input–output relations between questions and answers in a given round that Alice and Bob may utilize can be encoded in four single bits $\left(A_{0},A_{1},B_{0},B_{1}\right)$. In this notation, the number $A_{0}\in \left\{0;1\right\}$ is to be understood as the value that Alice will give as her answer *a* in a round in which she receives the input bit x=0. $A_{1}\in \left\{0;1\right\}$ is the value she will give as her output bit if she receives the input bit x=1. Similarly, the number $B_{0}\in \left\{0;1\right\}$ is to be understood as the value that Bob will give as his answer *b* in a round in which he receives the input bit y=0, whereas $B_{1}\in \left\{0;1\right\}$ denotes the output Bob will give in case of y=1. As shown in Table 1, each question regime has specific losing strategies.

It is easy to see which elementary strategies produce the best results when the question regime is unknown to both Alice and Bob. For example, always using the elementary strategy $\left(A_{0},A_{1},B_{0},B_{1}\right)=\left(1,1,1,1\right)$, Alice and Bob would win the round in the case of question regimes xy=00,01,10, but lose in the case of xy=11, so this strategy produces a win in 3 out of 4 regimes.

In general, the performance of an elementary strategy can be easily checked by observing that the number of question regimes *L*, in which Alice and Bob lose with the elementary strategy $\left(A_{0},A_{1},B_{0},B_{1}\right)$, can be computed as

(2) $$\begin{array}{c} L=A_{0}⊕B_{0}+A_{0}⊕B_{1}+A_{1}⊕B_{0}+\left(1-A_{1}⊕B_{1}\right). \end{array}$$

The validity of Equation (2) is easy to see, because in the question regimes 00,01, and 10, the goal of Alice and Bob is to generate a⊕b=0, while in the question regime 11 that Alice and Bob need to produce a⊕b=1. Running through the 16 possible elementary strategies shows that *L* can only take the values 1 or 3, noting that there is no elementary strategy that would produce L=0. Therefore, the best that Alice and Bob can do with an elementary strategy is to ensure that they only lose in one of four possible question regimes, and with $\left(A_{0},A_{1},B_{0},B_{1}\right)=\left(1,1,1,1\right)$, we have already found such a strategy. If all question regimes occur with equal likelihood during the game, this achieves a win in 75% of the rounds.

Therefore, the challenge given by the Verifier to Alice and Bob in this version of the game is *to produce a percentage of rounds of at least 75%+ν, for which Equation (1) holds.* Here, ν>0 is a threshold that is set before the game commences.

### 2.2. The Game with the Goal of Achieving a High *S*-Value

In a different presentation of the game, Alice and Bob need to produce a high *S*-value. The *S*-value is a statistical quantity derived from expectation values following the approach of Clauser–Horne–Shimony–Holt in [29]. In this version of the game, Alice and Bob receive questions x,y which are taken from the set {0,1}, to which they have to give answers a,b from the set {±1}.

Again, Alice and Bob work as a team that may pre-agree strategies but cannot communicate during the game. When all rounds have been played, the questions and answers will be put together and a list of quadruplets (a,b,x,y) results, which can be partitioned depending on the question regime. This gives a statistic P(ab|xy) for the product value ab∈{±1}, conditional upon each of the four different question regimes xy∈{00,01,10,11}. We can now compute expectation values for the product ab under these four question regimes xy by defining

$${\langle ab\rangle }_{xy}:=\sum_{ab}\;ab\;P\left(ab\;|\;xy\right).$$

We will assume that each question regime occurs at least once in the game, so that all four expectation values are well defined. Following [29], the combination of these four expectation values yields four *S*-values

(3) $$\begin{array}{ccc} S_{1} & = & \;\;\;{\langle ab\rangle }_{00}+{\langle ab\rangle }_{01}+{\langle ab\rangle }_{10}-{\langle ab\rangle }_{11}\;, \end{array}$$

(4) $$\begin{array}{ccc} S_{2} & = & \;\;\;{\langle ab\rangle }_{00}+{\langle ab\rangle }_{01}-{\langle ab\rangle }_{10}+{\langle ab\rangle }_{11}\;, \end{array}$$

(5) $$\begin{array}{ccc} S_{3} & = & \;\;\;{\langle ab\rangle }_{00}-{\langle ab\rangle }_{01}+{\langle ab\rangle }_{10}+{\langle ab\rangle }_{11}\;, \end{array}$$

(6) $$\begin{array}{ccc} S_{4} & = & -{\langle ab\rangle }_{00}+{\langle ab\rangle }_{01}+{\langle ab\rangle }_{10}+{\langle ab\rangle }_{11}\;. \end{array}$$

If Alice and Bob agree to always give the answers a=b=1, no matter what question they receive, they are able to generate $S_{i}=2$ for all i=1,2,3,4. However, it is unclear how higher *S*-values could be achieved. What makes winning the game difficult is the fact that, in the expression for any $S_{i}$, one of the correlations is subtracted from the other three. It is this feature of the $S_{i}$-values which precludes success using a simple strategy of always giving the same answer to the same question.

Therefore, the challenge given to Alice and Bob for this version of the game is *to produce an $S_{1}$-value of at least 2+η*. Here, η>0 is a threshold that is set before the game commences. Instead of $S_{1}$, one could also use one of the other values $S_{2},S_{3},S_{4}$ to formulate the challenge. As shown in Proposition 2 of [20], at most one of the four *S*-values may exceed 2. The well-known claim is that this game is impossible to win if an infinite number of rounds is played and if there are no additional resources, such as pairs of entangled particles, available to Alice and Bob.

### 2.3. On the Equivalence of the Two Game Descriptions

We want to show that the two versions of the game presented above are essentially the same. Conceptually, we therefore have to connect the probability of winning a single round to expectation values. To do this, we first note that, after a game over *N* rounds, the Verifier can break the complete list of quadruplets (a,b,x,y) into four lists by conditioning on specific question regimes xy. In general, these four lists will have different lengths, which we denote by $N_{xy}$, and we only know $N=N_{00}+N_{01}+N_{10}+N_{11}$. In each of those four sub-lists, the Verifier can count how often the product value ab was positive and how often it was negative. Let us assume that the Verifier uses the symbol $N_{00}^{+}$ for the number of rounds in which ab=+1 occurred in the sub-list with xy=00, and the symbol $N_{00}^{-}$ for the number of rounds in which ab=−1 occurred in the sub-list with xy=00. For the first expectation value term in Equation (3), we can therefore write

$$\begin{array}{c} {\langle ab\rangle }_{00}\;=\;\frac{N_{00}^{+}-N_{00}^{-}}{N_{00}}\;=\;1-2\frac{N_{00}^{-}}{N_{00}}\;=\;2\frac{N_{00}^{+}}{N_{00}}-1, \end{array}$$

because $N_{00}=N_{00}^{+}+N_{00}^{-}$. Analogous expressions hold for ${\langle ab\rangle }_{01},{\langle ab\rangle }_{10}$ and ${\langle ab\rangle }_{11}$. In particular, we have

$$\begin{array}{c} {\langle ab\rangle }_{11}\;=\;\frac{N_{11}^{+}-N_{11}^{-}}{N_{11}}\;=\;2\frac{N_{11}^{+}}{N_{11}}-1. \end{array}$$

From Equation (3), we therefore obtain

(7) $$\begin{array}{ccc} S_{1} & = & {\langle ab\rangle }_{00}+{\langle ab\rangle }_{01}+{\langle ab\rangle }_{10}-{\langle ab\rangle }_{11}\\ & = & 1-2\frac{N_{00}^{-}}{N_{00}}+1-2\frac{N_{01}^{-}}{N_{01}}+1-2\frac{N_{10}^{-}}{N_{10}}-\left(2\frac{N_{11}^{+}}{N_{11}}-1\right)\\ & = & 4-2\left(\frac{N_{00}^{-}}{N_{00}}+\frac{N_{01}^{-}}{N_{01}}+\frac{N_{10}^{-}}{N_{10}}+\frac{N_{11}^{+}}{N_{11}}\right). \end{array}$$

To connect the description of the game in Section 2.2, where the requirement is a,b∈{±1}, with the description in Section 2.1, where the answer values have to be in the set {0,1}, we start by denoting the answers in the description of Section 2.1 with an asterisk ∗, so that the requirement for winning a round by fulfilling Equation (1) reads $x\cdot y=a^{*}⊕\;b^{*}$. The transformation $a=2a^{*}-1$ and $b=2b^{*}-1$ bijectively maps the set {0,1} to the set {±1}. With this transformation, the 16 elementary strategies that Alice and Bob can use in a given round can be described as tuples $\left(A_{0},A_{1},B_{0},B_{1}\right)\in \left\{\pm 1\right\}\times \left\{\pm 1\right\}\times \left\{\pm 1\right\}\times \left\{\pm 1\right\}$. To relate this to the percentage of winning rounds, we define

$$L_{xy}:=\frac{\mathrm{number}\;\mathrm{of}\;\mathrm{losing}\;\;\mathrm{rounds}\;\mathrm{played}\;\mathrm{in}\;\mathrm{question}\;\mathrm{regime}\;xy}{\mathrm{total}\;\mathrm{number}\;\mathrm{of}\;\mathrm{round}\;\mathrm{played}\;\mathrm{in}\;\mathrm{question}\;\mathrm{regime}\;xy}.$$

In the question regime xy=00, losing a round is equivalent to $a^{*}⊕b^{*}=1$, which is equivalent to ab=−1. Therefore, $L_{00}=N_{00}^{-}/N_{00}$ is the percentage of losing rounds in question regime xy=00. Applying the same argument to the question regimes 01 and 10, we see that the losing percentages for these regimes are $L_{01}=N_{01}^{-}/N_{01}$ and $L_{10}=N_{10}^{-}/N_{10}$, respectively. For the question regime xy=11, the value of the product x·y is equal one, so Alice and Bob lose a round if and only if $a^{*}⊕b^{*}=0$. In this question regime, losing is equivalent to ab=+1, which gives the losing percentage $L_{11}=N_{11}^{+}/N_{11}$. From Equation (7), we therefore obtain

(8) $$\begin{array}{ccc} S_{1} & = & 4-2\left(\frac{N_{00}^{-}}{N_{00}}+\frac{N_{01}^{-}}{N_{01}}+\frac{N_{10}^{-}}{N_{10}}+\frac{N_{11}^{+}}{N_{11}}\right)\\ & = & 4-2(L_{00}+L_{01}+L_{10}+L_{11}). \end{array}$$

The last equation shows that maximizing the value of $S_{1}$ is tantamount to minimizing the losing percentages in the question regimes.

As discussed in Section 2.1, with every elementary strategy Alice and Bob face at least one losing question regime. Using the same elementary strategy in all game rounds and assuming that the Quiz Master uses all question regimes during the game, this implies that $L_{00}+L_{01}+L_{10}+L_{11}⩾1$ and hence $S_{1}⩽2$ by Equation (8).

The situation becomes more involved if Alice and Bob switch between different elementary strategies during the game. In some rounds, they may be lucky to have chosen the right elementary strategy to produce a win. Intuitively, when the game is played over many rounds and all question regimes come up unpredictably with non-negligible frequency, then one would expect the chances of being lucky enough to produce $L_{00}+L_{01}+L_{10}+L_{11}<1$ to diminish. If Alice and Bob randomly switch between elementary strategies and if the Quiz Master randomly switches between the four question regimes, we expect equal percentages of losing rounds in all question regimes, i.e., $L_{00}=L_{01}=L_{10}=L_{11}=:L$, over the long run. Under this additional assumption, Equation (8) reads $S_{1}=4-8L$, which shows that $S_{1}=2$ is equivalent to L=25%, so $S_{1}=2$ translates to a winning percentage of 75%, which makes the challenges given at the end of Section 2.1 and Section 2.2 equivalent (with η=8ν).

However, winning is subject to statistical fluctuations, and Alice and Bob may try to benefit from the unequal frequencies of question regimes if they are aware of them. In the next chapter, we will give bounds for the probability of winning by pure luck if the question regimes occur with unequal probabilities.

## 3. Bounds for the Likelihood of Being Lucky

The well-known claim is that the CHSH game is impossible to win if the questions are asked randomly and Alice and Bob have no additional (non-local) resources, such as pairs of entangled particles, at their disposal. In fact, this is the reason which makes CHSH games useful tools for an explanation of the power of entanglement.

To make the claim true, we first have to insist on ruling out cheats, in which Alice and Bob may prompt some of the questions themselves or acquire knowledge about the question regime they will receive during the game. Such cheats could have consequences which are quantitatively equal to a violation of locality [19,30]. Going forward, we insist on the *free* and *independent* generation of all questions, although we do not insist that all question regimes occur with equal likelihood.

The objections that Alice and Bob may win by luck or clever play still have to be dealt with, and we will deal with this now. In this, one needs to keep in mind that they can play in different ways and “being lucky“ is a difficult term, as the effect of luck may in principle depend on how they play the game. In particular, Alice and Bob can:

(i) Draw up a list, which specifies in advance which elementary strategy they will use in a game round only based on the question posed in this round;
(ii) Create a deterministic algorithm that determines how they will answer in a given round based on the information about all questions they have received so far locally;
(iii) Use the information about all answers they have given so far locally and all questions they have received so far locally as an input to an algorithm, which may use classical local independent randomization procedures while the game is ongoing, to generate their answers;
(iv) Determine how they will answer in a given round using a *momentary hunch* (i.e., *gut feeling*) while the game is ongoing.

These four approaches will be discussed to determine the effect of luck. Randomness may lie in the generation of the questions x,y; however, under Approaches (iii) and (iv), it may also lie in the generation of the answers. We will now argue that Approaches (i)–(iii) result in the answers being drawn independently from two N×2 tables filled with numbers of plus or minus one (i.e., from two “*spreadsheets*”).

In Approach (i), by definition, Alice will have a list of pairs $\left(A_{0},A_{1}\right)\in \left\{\pm 1\right\}\times \left\{\pm 1\right\}$ to determine her answers in each round and Bob will have another list of pairs $\left(B_{0},B_{1}\right)\in \left\{\pm 1\right\}\times \left\{\pm 1\right\}$ to determine his answers. The output bit *a* given by Alice in round *n* of the game is read out from row *n* of her list as

(9) $$\begin{array}{c} a:=\left\{\begin{array}{cc} A_{0} & \mathrm{if}\;x=0,\\ A_{1} & \mathrm{if}\;x=1, \end{array}\right. \end{array}$$

and the output bit *b* given by Bob in round *n* is determined from row *n* of his list by

(10) $$\begin{array}{c} b:=\left\{\begin{array}{cc} B_{0} & \mathrm{if}\;y=0,\\ B_{1} & \mathrm{if}\;y=1. \end{array}\right. \end{array}$$

Prior to the start of the game, Alice and Bob may create and discuss their lists together and Alice may even take a copy of Bob’s list into her room, but that will not make a difference as she is only allowed to have local knowledge during the game. The important part is that we have counter-factual definiteness for the answers of Alice and Bob as they are read off from a table that covers all situations.

Approach (ii) generalizes Approach (i), but can still be formalized by pre-agreeing upon a set of elementary strategies for each possible path the game may take up to a given round *n*. Written formally, in each round, Alice receives a question and gives an answer so that the locally available information for her in round *n* consists of a question list $x_{1}x_{2}\ldots x_{n}$ and a list of answers $a_{1}a_{2}\ldots a_{n-1}$ which she has already given. Based on this information, she has to determine her answer $a_{n}$ for the current round *n*, in which question $x_{n}$ was posed. In principle, assuming sufficient memory, Alice and Bob could prepare a game over a total of *N* rounds by pre-agreeing upon a large collection of tables to formalize their strategy under Approach (ii). For example, based on the information up to and including round n−1, there are $4^{n-1}$ possible combinations of (global) question regimes that could have been used, out of which Alice can differentiate $2^{n-1}$ based on her local knowledge. To determine her next answer $a_{n}$, she would pick her table corresponding to the history observed by her so far locally and read out row *n*. If that row reads $\left(A_{0},A_{1}\right)$, she will use the current question $x_{n}$ and give her answer using Equation (9) with $x:=x_{n}$. Bob will work analogously on his side. Again, the key point is that assuming the existence of a collection of tables corresponds to counter-factual definiteness as far as the answers of Alice and Bob are concerned. The questions they will receive may not be pre-determined, but Alice and Bob can state how they would have answered if they would have received different questions during the game. At the end of the day, the answers will have been generated by independent draws from two N×2 tables. Conceptually, two N×2 tables could evidently be seen as N×4 tables, as in [26], but if Alice and Bob do not reveal their strategies to each other, then neither of the players has access to the full N×4 tables. If one wishes to discuss “realism“, the collection of tables would correspond to elements of reality that could be read out by asking the right sequence of questions despite the fact that not all tables are read out in an actual game.

Approach (iii) generalizes Approach (ii), and now, the algorithm may be thought of as a collection of tables corresponding to a locally observed history, but allowing several tables that correspond to the same history. Out of these tables, Alice will pick the table she will use in the current round by casting a classical die in her room, where the number of sides equals the number of her tables that is consistent with her locally observed history. This randomization shows Alice which table to use in the current round. If, in that table, she finds the entry $\left(A_{0},A_{1}\right)$ in row *n*, she will use the current question $x_{n}$ and give her answer using Equation (9) with $x:=x_{n}$. Bob would proceed in an analogous way and the important assumption is that Bob’s die is cast independently from Alice’s die.

For Approaches (i)–(iii), the answers can be regarded as being generated from two N×2 tables by independent draws. This allows the derivation of the CHSH inequality: We start by observing that, when a combined row $\left(A_{0},A_{1},B_{0},B_{1}\right)$ is created from the two tables of numbers ±1, then we always obtain

$$A_{0}B_{0}+A_{0}B_{1}+A_{1}B_{0}-A_{1}B_{1}=A_{0}\left(B_{0}+B_{1}\right)+A_{1}\left(B_{0}-B_{1}\right)\in \left\{\pm 2\right\}.$$

Taking averages over all rounds, we conclude with the usual Bell-type argument

(11) $$\begin{array}{c} S_{1}={\langle ab\rangle }_{00}+{\langle ab\rangle }_{01}+{\langle ab\rangle }_{10}-{\langle ab\rangle }_{11}⩽2. \end{array}$$

We remark that moving from individual game rounds to averages and expectation values does require additional thoughts about the convergence of relative frequencies, because the answers given during the game do not have to be independent over the game rounds. We refer to [31] for details and give numerical examples in Section 4.

Approach (iv) is outside the scope of mathematical procedures because humans determining answers “on a momentary hunch“ seems to be a concept that is impossible to formalize. In this context, some people would treat Approach (iv) just as a form of randomness and automatically subsume it under Approach (iii), but it is important to keep in mind that such a treatment is an assumption based on certain philosophic positions. While the hunch of Alice experienced in round *n* can be influenced by the history of questions observed by her previously, some people would argue that this history alone does not allow a comprehensive description of her possibilities. They might argue that the local future of Alice or non-local connections to Bob cannot be ruled out a priori in the creation of a hunch. To see whether something such as Approach (iv) can provide more than Approach (iii) in terms of winning a CHSH game without having entanglement as a resource, actual experiments would have to be performed, which can only naturally occur over a finite number of rounds. The burden of proof certainly lies on those, which claim that Approach (iv) can yield more than Approach (iii) and bounds for the likelihood of winning by pure luck are given below.

Generally, it is believed that Alice and Bob cannot win the CHSH game unless they have additional non-local resources, such as pairs of entangled particles. However, if only a finite number of rounds *N* of the game are played, Alice and Bob may be declared winners, because the losing percentages that were actually realized may happen to be such that $S_{1}⩾2+\eta$ occurs in Equation (8) by pure chance. If Alice and Bob switch between elementary strategies between the rounds, e.g., under Approach (iii), then after *N* rounds, they may have been lucky to have picked many winning ones even if the four-question regimes xy were selected at random and with equal probability. We want to determine how much luck is needed for this to happen. Formulated more precisely, we want to prove statistical bounds for the probability that winning happens by pure luck. Mathematically, such proofs depend on so-called concentration inequalities, which are bounds for the probability that a sum of random variables deviates from its expected value, and by a certain amount. In this area, mathematical contributions were made by Hoeffding, Bernstein, Chernoff, Azuma, Bentkus, McDiarmid, Combes, and others, and many technical formulations exist. We will use Hoeffding inequalities [32] and an extension of McDiarmid inequalities [33], giving a self-contained proof of the required results of the former for the completeness of presentation.

In what follows, it is important to note that the number of rounds *N* has to be fixed in advance and agreed between the players and the Verifier. If the number of rounds is not fixed beforehand, then one can be tempted to play until a desired level of violation has been reached. Such an approach can invalidate the entire statistical analysis, as can be seen in [28], because the probability distribution for the maximum of a stochastic process is different from the probability distribution of that process.

### 3.1. Bounds Based on Hoeffding Inequalities

As shown by Gill as Theorem 1 in [26], when the observed answers are completely selected at random by fair, i.e., 50:50, coin tosses, the probability of generating an $S_{1}$-values above 2 is limited by

(12) $$\begin{array}{c} \mathrm{Pr}\left\{S_{1}⩽2+\eta \right\}⩾1-8e^{-N{\left(\eta /16\right)}^{2}} \end{array}$$

for any η⩾0. Mathematically, the proof of that bound in [26] is essentially a repeated application of Hoeffding’s inequalities [32]. The right-hand side of the inequality requires a larger number of rounds to give meaningful bounds as shown in column (a) of Table 2.

The derivation of Equation (12) stated above that is given in [26] is based on assuming that starting from a spreadsheet containing a table with *N* rows and 4 columns, fully populated with entries of ±1, for each row, “*two fair coins are tossed independently of one another, independently over all the rows*”. Depending on the outcomes of the two coin tosses for a round, one value from the first two columns and one value from the last two columns is observed in the round corresponding to the specific row. While this gives a clear picture of the assumed randomness, it creates an operational problem, because no one in the CHSH game may have this full spreadsheet: Alice and Bob can work with different lists which they did not share and the Verifier only has access to the list of answers that were actually given.

We will now give a self-contained proof for a bound that is stronger than Equation (12) and that does not require the assumption of a fair coin toss for the selection of the question regime. Relaxing this assumption is important, because the four question regimes {00,01,10,11} may occur with a somewhat unequal likelihood in physical Bell experiments given biases of random number generators. In a game between humans, in which a Quiz Master is free to choose the questions, the equal likelihood of question regimes cannot be assumed a priori. In macroscopic situations, the available table of observations may not necessarily be assumed to contain the four regimes with equal probability. For example, if in the macroscopic application described in [19], large orders occur rarely and there are few rumors, regime xy=00 will be prevalent. In this case, the first expectation term in the expression $S_{1}={\langle ab\rangle }_{00}+{\langle ab\rangle }_{01}+{\langle ab\rangle }_{10}-{\langle ab\rangle }_{11}$ will be computed from the observations of ab over many rounds, while the other three expectation values will result from the observations over relatively few rounds. In such a case of highly asymmetric frequency for the different regimes, randomness may have a stronger influence on the $S_{1}$ value. In an extreme case, where each of the three regimes {01,10,11} occurs only once during the game rounds and chance happens to produce ${\langle ab\rangle }_{01}=1,\;{\langle ab\rangle }_{10}=1,\;{\langle ab\rangle }_{11}=-1$, we obtain $S_{1}={\langle ab\rangle }_{00}+3$.

Therefore, with the subsequent Theorem 1, we will extend the work in [26] to situations where the four regimes may occur with different probabilities. We will, however, stick with the key idea of the proof made by Gill, i.e., a repeated application of Hoeffding’s inequalities. Before stating and proving Theorem 1, we start by proving Hoeffding’s inequality by elementary means, to provide the reader with a fully self-contained presentation.

**Proposition** **1.** *Let $X_{1},\cdots ,X_{N}$ be independent random variables with values $X_{i}$ in the interval [0;1]. Then, for all δ>0, we have*

(13) $$\begin{array}{c} Pr\left\{\frac{X_{1}+\cdots +X_{N}}{N}>\mu +\delta \right\}⩽\exp\left(-2N\delta^{2}\right) \end{array}$$

*and*

(14) $$\begin{array}{c} Pr\left\{\frac{X_{1}+\cdots +X_{N}}{N}<\mu -\delta \right\}⩽\exp\left(-2N\delta^{2}\right) \end{array}$$

*with $\mu :=E[\left(X_{1}+\cdots +X_{N}\right)/N]$.*

**Proof.**  To give a self-contained proof, we start by making the following observation: if *Y* is a random variable with non-negative values, then for each positive real number *a*, we may trivially write

$$Y\geq a1_{\left\{Y>a\right\}}$$

using the indicator function $1_{\left\{\ldots \right\}}$. Taking the expectation value is a monotonic and linear operation, so

$$E\left[Y\right]\geq E\left[a1_{\left\{Y>a\right\}}\right]=aE\left[1_{\left\{Y>a\right\}}\right]=a\;Pr\left\{Y>a\right\}$$

follows, which proves the so-called Markov inequality

(15) $$\begin{array}{c} Pr\left\{Y>a\right\}\leq \frac{1}{a}E\left[Y\right]. \end{array}$$

Second, we observe that, for arbitrary real numbers *r* and each constant c∈[0;1], we have

(16) $$\begin{array}{c} 1+\left(e^{r}-1\right)c\leq e^{rc+r^{2}/8}, \end{array}$$

which can be shown as follows: consider the logarithm of the left-hand side and define the function $f\left(r\right):=\ln(1+\left(e^{r}-1\right)c)$. This function is twice continuously differentiable with

$$f^{\prime }\left(r\right)=\frac{e^{r}c}{1+(e^{r}-1)c}=\frac{c}{c+\left(1-c\right)e^{-r}}\;\;\;\mathrm{and}\;\;f^{\prime \prime }\left(r\right)\leq \frac{1}{4}.$$

Because of f(0)=0 and $f^{\prime }\left(0\right)=c$, the second-order Taylor expansion of *f* yields

$$f\left(r\right)=f\left(0\right)+rf^{\prime }\left(0\right)+\frac{1}{2}r^{2}f^{\prime \prime }\left(r\theta \right)\leq rc+\frac{1}{8}r^{2},$$

with some θ∈[0;1], which proves Equation (16).

Third, we observe that for all real numbers *r*, the exponential function $x\to e^{rx}$ is convex and hence, for all x∈[0;1], we have

$$e^{rx}\leq xe^{r}+\left(1-x\right)e^{0}=1+\left(e^{r}-1\right)x.$$

Thus, for each random variable $X_{i}$, we obtain

$$e^{rX_{i}}\leq 1+\left(e^{r}-1\right)X_{i}\;\;,$$

which leads to

$$E\left[e^{rX_{i}}\right]\leq 1+\left(e^{r}-1\right)E\left[X_{i}\right]\overset{Equation\;(16)}{\leq }e^{rEX_{i}+r^{2}/8}.$$

This directly implies the so-called Hoeffding Lemma

(17) $$\begin{array}{c} E\left[e^{r(X_{i}-EX_{i})}\right]\leq e^{r^{2}/8}. \end{array}$$

After these three preparatory steps, we can make the following estimate, which holds for all positive real numbers *r*

$$\begin{array}{ccc} Pr\left\{\frac{X_{1}+\cdots +X_{N}}{N}>\mu +\delta \right\} & = & Pr\left\{r\left(X_{1}+\cdots +X_{N}\right)>rN\left(\mu +\delta \right)\right\}\\ & = & Pr\left\{e^{r(X_{1}+\cdots +X_{N})-rN\mu }>e^{r\delta N}\right\}\\ & \overset{Equation\;(15)}{\leq } & e^{-r\delta N}E\left[e^{r(X_{1}+\cdots +X_{N})-rN\mu }\right]\\ & = & e^{-r\delta N}E\left[\prod_{i=1}^{N}e^{r(X_{i}-EX_{i})}\right]\\ & \overset{indep.}{=} & e^{-r\delta N}\prod_{i=1}^{N}E\left[e^{r(X_{i}-EX_{i})}\right]\\ & \overset{Equation\;(17)}{\leq } & e^{-r\delta N}\prod_{i=1}^{N}e^{r^{2}/8}=e^{-r\delta N}e^{Nr^{2}/8}=e^{(r^{2}-8r\delta )N/8}\\ & = & \exp\left(({\left(r-4\delta \right)}^{2}-16\delta^{2})N/8\right). \end{array}$$

The exponent in the last expression is minimized with the choice r:=4δ. This proves Equation (13).

Knowing that Equation (13) holds true, it is easily seen that Equation (14) also holds true: we only have to define $Y_{i}:=1-X_{i}$ which gives a collection of independent random variables $Y_{1},\ldots ,Y_{N}$ with values in the interval [0;1] to which Equation (13) can be applied. □

Now, we can prove

**Theorem** **1.** *Assume that, for each round of the N rounds of the CHSH game one of the four question regimes, xy is selected at random with a constant probability $p_{xy}$ independently of the selection for the other rounds of the game. Further assume that there are N×2 tables of numbers ±1, from which the answers of Alice and Bob in each given round are determined as described earlier under the Approaches (i), (ii) or (iii) leading to the expectation of $S_{1}⩽2$.*

*Then, for any positive number η, the probability of observing an $S_{1}$-value above 2+η at the end of the CHSH game is limited by*

$$\begin{array}{c} Pr\{S_{1}^{\mathrm{obs}}⩽2+\eta \}⩾1-q, \end{array}$$

*where the bound q is given by*

(18) $$\begin{array}{c} q:=4\exp\left(-2N\delta^{2}\right)+\sum_{xy}\exp\left(-2N\left(p_{xy}-\delta \right){\left(\eta /8\right)}^{2}\right). \end{array}$$

*In this bound, the number δ>0 can be freely chosen to minimize the value of q.*

**Proof.**  The proof uses arguments similar to those made by Gill in [26]. We use the notation introduced in Section 2.3 and added the superscript “*(obs)*” to those numbers that are calculated from the question and answer pattern that was actually observed in the CHSH game.

Let two positive numbers η and δ be given and let us write $\tilde{\delta }:=\eta /8$. Applying Equation (14) for each question regime xy=00,01,10,11, we see that the probability of a deviation of the sample mean from the theoretical probability $p_{xy}$ of the question regime is bounded by

(19) $$\begin{array}{c} \mathrm{Pr}\left\{\frac{N_{xy}^{\mathrm{obs}}}{N}⩽p_{xy}-\delta \right\}⩽\exp\left(-2N\delta^{2}\right). \end{array}$$

Thus, ignoring the union of all sets for which (19) does not hold, we have

(20) $$\begin{array}{c} N_{xy}^{\mathrm{obs}}>N\left(p_{xy}-\delta \right)\;\;\mathrm{for}\;\mathrm{all}\;xy=00,01,10,11, \end{array}$$

except possibly on a set that has a probability of at most $4\exp(-2N\delta^{2})$.

Conditional on $N_{xy}^{\mathrm{obs}}$ observations being made in question regime xy, we have to compare the observed sample mean for a positive product $N_{xy}^{\mathrm{obs},+}/N_{xy}^{\mathrm{obs}}$ with its expected value $N_{xy}^{+}/N_{xy}$. Again, applying Hoeffding’s inequalities [32,34], we obtain the bound

$$\begin{array}{c} \mathrm{Pr}\left\{\frac{N_{xy}^{\mathrm{obs},+}}{N_{xy}^{\mathrm{obs}}}⩾\frac{N_{xy}^{+}}{N_{xy}}+\tilde{\delta }\right\}⩽\exp\left(-2N_{xy}^{\mathrm{obs}}{\tilde{\delta }}^{2}\right),\;\;\mathrm{for}\;\mathrm{all}\;xy=00,01,10 \end{array}$$

and similarly

$$\begin{array}{c} \mathrm{Pr}\left\{\frac{N_{11}^{\mathrm{obs},-}}{N_{11}^{\mathrm{obs}}}⩾\frac{N_{11}^{-}}{N_{11}}+\tilde{\delta }\right\}⩽\exp\left(-2N_{11}^{\mathrm{obs}}{\tilde{\delta }}^{2}\right). \end{array}$$

Using (20), the upper bound for these probabilities can be dominated by

$$\begin{array}{c} \exp\left(-2N_{xy}^{\mathrm{obs}}{\bar{\delta }}^{2}\right)⩽\exp\left(-2N\left(p_{xy}-\delta \right){\bar{\delta }}^{2}\right). \end{array}$$

So, except on a small set, the observed frequency of each question regime and the observed frequency of a positive or negative product ab in each question regime may not deviate too far from their overall mean. As shown in Section 2.3, we have

$$\begin{array}{ccc} {\langle ab\rangle }_{00}\; & = & \;\;\;\frac{N_{00}^{+}-N_{00}^{-}}{N_{00}}\;=\;2\frac{N_{00}^{+}}{N_{00}}-1,\\ {\langle ab\rangle }_{01}\; & = & \;\;\;\frac{N_{01}^{+}-N_{01}^{-}}{N_{01}}\;=\;2\frac{N_{01}^{+}}{N_{01}}-1,\\ {\langle ab\rangle }_{10}\; & = & \;\;\;\frac{N_{10}^{+}-N_{10}^{-}}{N_{10}}\;=\;2\frac{N_{10}^{+}}{N_{10}}-1,\\ -{\langle ab\rangle }_{11}\; & = & -\frac{N_{11}^{+}-N_{11}^{-}}{N_{11}}\;=\;2\frac{N_{11}^{-}}{N_{11}}-1. \end{array}$$

These equations remain true, when the reference is made to observed quantities only, i.e., when ${\langle ab\rangle }_{xy}$ is replaced by ${\langle ab\rangle }_{xy}^{\mathrm{obs}}$ and $N_{xy}$ by $N_{xy}^{\mathrm{obs}}$, etc. Therefore, we conclude by using (11) that the inequality

$$\begin{array}{ccc} S_{1}^{\mathrm{obs}} & = & {\langle ab\rangle }_{00}^{\mathrm{obs}}+{\langle ab\rangle }_{01}^{\mathrm{obs}}+{\langle ab\rangle }_{10}^{\mathrm{obs}}-{\langle ab\rangle }_{11}^{\mathrm{obs}}\\ & ⩽ & {\langle ab\rangle }_{00}+{\langle ab\rangle }_{01}+{\langle ab\rangle }_{10}-{\langle ab\rangle }_{11}+8\tilde{\delta }=S_{1}+8\tilde{\delta }\\ & ⩽ & 2+8\tilde{\delta }=2+\eta \end{array}$$

has to hold, except possibly on a set with a probability of no more than

$$\begin{array}{c} 4\exp\left(-2N\delta^{2}\right)+\sum_{xy}\exp\left(-2N\left(p_{xy}-\delta \right){\left(\eta /8\right)}^{2}\right). \end{array}$$

Note that, in the last expression, we are free to choose any positive δ and the sum has to be taken over all four question regimes xy=00,01,10,11. □

For given parameters $N,p_{xy},\eta$ the value for δ, which minimizes *q* in Equation (18) and hence gives the best bound, can be found numerically, for example, by solving 0=dq/dδ. Performing the minimization, for example, for the case of equally likely question regimes $p_{xy}=0.25$ with N=5000 and η=0.5, an optimal value of δ≈0.03361 is found. With this value, Theorem 1 provides the bound $\mathrm{Pr}\{S_{1}^{\mathrm{obs}}⩽2.5\}⩾99.90\%$, which is shown in Column (b) in the last row of Table 2.

### 3.2. Bounds Based on an Extended McDiarmid Inequality

To the extent that one is interested in some ideas behind the mathematics needed for more advanced bounds, an extension of the McDiarmid inequality can be of interest. In the proof of Theorem 1, Proposition 1 is applied separately to each of the four expectation values that occur in the definition of $S_{1}$ in Equation (3). The probabilities of the four exception sets, on which the observed averages deviate substantially from the theoretical expectation value, are then simply added. However, the exception sets overlap and cancellations may be expected to occur. In this section, we will work directly with the definition of the $S_{1}$-value as a linear combination of random variables with values of ±1. We will also use a different concentration inequality, namely an extension of the McDiarmid inequality, to arrive at a powerful bound for the probability of winning the CHSH game by pure chance.

McDiarmid’s inequalities build on the work of Hoeffding and Azuma and give concentration inequalities for situations in which differences for the evaluating function are bounded, i.e., satisfy a Lipschitz condition, as can be seen in [35]. They were extended in [33] by Combes to cover situations in which the differences are not bounded everywhere, but only on a set that has a high probability. Of course, better bounds usually come at the price of higher complexity, so we will not attempt a self-contained presentation of results, but rather summarize McDiarmid and Combes’ result in the following

**Proposition** **2.** *Let $X_{1},\ldots ,X_{N}$ be sets. Define $X:=X_{1}\times \cdots \times X_{N}$ and let Y be a subset of X. Let f:X→R be a function that assigns a real number f(x) to every $x=(x_{1},\ldots ,x_{N})\in X$. Assume that, on the set Y, the function f has bounded differences, i.e., there are constants $c_{1},\ldots ,c_{N}$ such that*

(21) $$\begin{array}{c} |f\left(x\right)-f\left(x^{\prime }\right)|⩽c_{i}\;\;\mathrm{for}\;\mathrm{all}\;\left(x,x^{\prime }\right)\in Y\times Y\;\mathrm{with}\;x_{j}=x_{j}^{\prime }\;\;\mathrm{for}\;j\neq i. \end{array}$$

*Let $X_{1},\ldots ,X_{N}$ be independent random variables with values $X_{i}$ in the set $X_{i}$ and let us write $X:=(X_{1},\ldots ,X_{N})$ as well as m:=E[f(X)|X∈Y]. Then, for all η⩾0, we have*

(22) $$\begin{array}{c} Pr\left\{f\left(X\right)-m⩾\eta \right\}⩽p+\exp\left(-\frac{2\max{\left(\eta -p\sum_{i=1}^{N}c_{i},0\right)}^{2}}{\sum_{i=1}^{N}c_{i}^{2}}\right) \end{array}$$

*with p:=Pr{X∉Y}.*

**Proof.**  See [33]. □

While a proof cannot be given here, Proposition 2 is not so difficult to understand intuitively: if one has a set Y on which the function is well behaved, i.e., small random fluctuations in the inputs do not create a big difference in its value, then one can estimate the probability for a large deviation from the mean by an exp(−⋯) term, vaguely reminiscent of Hoeffding’s inequality. Outside the set, nothing can be said, but that is not a problem as long as the probability of being in Y can be made very high by playing a sufficient number of rounds.

Proposition 2 can be applied to the CHSH game in the following way: under Approach (iii), there are N×2 spreadsheets that specify how Alice and Bob will answer. Knowing all questions for all rounds, $S_{1}$ can be computed via Equation (3), and this procedure for obtaining $S_{1}$ plays the role of the function *f* in Proposition 2. The random variable $X_{i}$ determines which question regime applies in round *i*, so it can take any of the four values in the set $X_{i}:=\left\{00,01,10,11\right\}$. The assumption that, in each round of the game, one of the four question regimes xy is selected at random with a probability of $p_{xy}$ independently from the selection for the other rounds of the CHSH game, gives the independence property for the random variables $X_{1},\ldots ,X_{N}$.

To show that computing $S_{1}$ has bounded differences on an appropriately defined set, we first note that the expectation values ${\langle ab\rangle }_{xy}$ in (3) are taken over $N_{xy}$ observations. The numbers $N_{xy}$, which were introduced in Section 2.3 count how often the question regime xy has occurred in the CHSH game. Formally, these numbers $N_{xy}$ result via

$$\begin{array}{ccc} N_{xy} & = & \sum_{i=1}^{N}1_{\left\{X_{i}\;=\;xy\right\}} \end{array}$$

from the random variables $X_{i}$. To show that $S_{1}$ has bounded differences on a subset of X, we define such a subset Y by making sure that inside it, all four question regimes occur sufficiently often. This is achieved by

$$\begin{array}{c} Y:=X\setminus (\left\{N_{00}⩽K+1\right\}\cup \left\{N_{01}⩽K+1\right\}\cup \left\{N_{10}⩽K+1\right\}\cup \left\{N_{11}⩽K+1\right\}), \end{array}$$

for some natural number *K*. We compute

Pr{Nxy⩽K+1}=∑k=0K+1Nkpxyk(1−pxy)N−k

for all xy=00,01,10,11, which allows the following crude upper bound for its probability

(23) Pr{X∉Y}=Pr⋃xy{Nxy⩽K+1}⩽∑xyPr{Nxy⩽K+1}=∑xy∑k=0K+1Nkpxyk(1−pxy)N−k.

An exact computation of Pr{X∉Y} would be possible by using the properties of the multinomial distribution, as can be seen in [36,37], but we want to avoid such technical arguments.

As we will show in the subsequent proof, the function $S_{1}:X\to \left[-4;+4\right]$ has bounded differences, i.e., obeys Equation (21), on the set Y. We will show below that, when we change the outcome of just one random variable $X_{i}$, then the resulting change in the value of $S_{1}$ is bounded by $c_{i}=4/K$. On this basis, an application of Proposition 2 yields the following

**Theorem** **2.** *Assume that, for each round of N rounds of the CHSH game, one of the four question regimes xy is selected at random with a constant probability of $p_{xy}$, independently of the selection for the other rounds of the game. Further assume that there are N×2 tables of numbers ±1, from which the answers of Alice and Bob in each given round are determined as described earlier under Approaches (i), (ii) or (iii) leading to the expectation of $S_{1}⩽2$.*

*Then, for any positive number η, the probability of observing an $S_{1}$-value above 2+η after N rounds of the CHSH game is limited by*

$$\begin{array}{c} Pr\{S_{1}^{\mathrm{obs}}⩽2+\eta \}⩾1-q \end{array}$$

*with*

(24) q:=p+exp−2max(η−4pN/K,0)216NK2,p:=∑xy∑k=0K+1Nkpxyk(1−pxy)N−k

*for all K<N/4.*

**Proof.**  We apply Proposition 2 with $f\left(X\right)=S_{1}^{\mathrm{obs}}$. The derivation of the expectation $S_{1}⩽2$ under Approach (i), (ii) or (iii) also works on the subset Y, which implies

$$\begin{array}{c} m=E[f(X)|X\in Y]⩽2. \end{array}$$

With the definition $\tilde{p}:=Pr\left\{X\notin Y\right\}$, Proposition 2 now provides the estimate

$$\begin{array}{ccc} Pr\{S_{1}^{\mathrm{obs}}>2+\eta \} & = & Pr\{f(X)-2>\eta \}⩽Pr\{f(X)-m>\eta \}\\ & ⩽ & \tilde{p}+\exp\left(-\frac{2\max{\left(\eta -\tilde{p}\sum_{i=1}^{N}c_{i},0\right)}^{2}}{\sum_{i=1}^{N}c_{i}^{2}}\right). \end{array}$$

From this, we obtain the claimed Equation (24) with $c_{i}:=4/K$ for all *i*, because we know $\tilde{p}⩽p$ from Equation (23).

To complete the proof of Theorem 2, it therefore only remains to be shown that the function $S_{1}:X\to \left[-4;+4\right]$ obeys Equation (21) with constants $c_{i}=4/K$ on the set Y that was defined above. To prove this, let us, to avoid notation that is too complex, for example, look at i=42 and assume that $x_{42}$ changes its value from $x_{42}=00$ to $x_{42}^{\prime }=01$ while the other variable $x_{1},\ldots ,x_{41},x_{43},\ldots ,x_{N}$ remains unchanged. This leads to a change from $S_{1}$ to $S_{1}^{\prime }$, so we need to estimate the magnitude of this change on the set Y. As a result of this change from $x_{42}$ to $x_{42}^{\prime }$, the number $N_{00}$ changes to $N_{00}^{\prime }=N_{00}-1$ and $N_{01}$ changes to $N_{01}^{\prime }=N_{01}+1$, while $N_{10}$ and $N_{11}$ remain unaffected. As a consequence, the difference between $S_{1}$ and $S_{1}^{\prime }$ can be written as

(25) $$\begin{array}{ccc} S_{1}-S_{1}^{\prime } & = & \frac{N_{00}^{+}-N_{00}^{-}}{N_{00}}+\frac{N_{01}^{+}-N_{01}^{-}}{N_{01}}-\left(\frac{{\left(N_{00}^{+}\right)}^{\prime }-{\left(N_{00}^{-}\right)}^{\prime }}{N_{00}^{\prime }}+\frac{{\left(N_{01}^{+}\right)}^{\prime }-{\left(N_{01}^{-}\right)}^{\prime }}{N_{01}^{\prime }}\right)\\ & = & \left(\frac{N_{00}^{+}-N_{00}^{-}}{N_{00}}-\frac{{\left(N_{00}^{+}\right)}^{\prime }-{\left(N_{00}^{-}\right)}^{\prime }}{N_{00}-1}\right)+\left(\frac{N_{01}^{+}-N_{01}^{-}}{N_{01}}-\frac{{\left(N_{01}^{+}\right)}^{\prime }-{\left(N_{01}^{-}\right)}^{\prime }}{N_{01}+1}\right)\\ & = & \frac{\left(N_{00}-1\right)\left(N_{00}^{+}-N_{00}^{-}\right)-N_{00}\left({\left(N_{00}^{+}\right)}^{\prime }-{\left(N_{00}^{-}\right)}^{\prime }\right)}{N_{00}\left(N_{00}-1\right)}\\ & & +\frac{\left(N_{01}+1\right)\left(N_{01}^{+}-N_{01}^{-}\right)-N_{01}\left({\left(N_{01}^{+}\right)}^{\prime }-{\left(N_{01}^{-}\right)}^{\prime }\right)}{N_{01}\left(N_{01}+1\right)}\\ & = & \frac{N_{00}\left(N_{00}^{+}-{\left(N_{00}^{+}\right)}^{\prime }\right)-N_{00}\left(N_{00}^{-}-{\left(N_{00}^{-}\right)}^{\prime }\right)-\left(N_{00}^{+}-N_{00}^{-}\right)}{N_{00}\left(N_{00}-1\right)}\\ & & +\frac{N_{01}\left(N_{01}^{+}-{\left(N_{01}^{+}\right)}^{\prime }\right)-N_{01}\left(N_{01}^{-}-{\left(N_{01}^{-}\right)}^{\prime }\right)+\left(N_{01}^{+}-N_{01}^{-}\right)}{N_{01}\left(N_{01}+1\right)} \end{array}$$

We introduce the following definitions for the change in the number of winners and losers in each question regime xy when we move from $x_{42}$ to $x_{42}^{\prime }$, namely

$$\begin{array}{ccc} \Delta_{xy}^{+} & := & N_{xy}^{+}-{\left(N_{xy}^{+}\right)}^{\prime },\;\;\Delta_{xy}^{-}\;:=\;N_{xy}^{-}-{\left(N_{xy}^{-}\right)}^{\prime }. \end{array}$$

Using this notation, we can write (25) as

$$\begin{array}{ccc} S_{1}-S_{1}^{\prime } & = & \frac{1}{N_{00}-1}\left(\Delta_{00}^{+}-\Delta_{00}^{-}-\frac{N_{00}^{+}-N_{00}^{-}}{N_{00}}\right)+\frac{1}{N_{01}+1}\left(\Delta_{01}^{+}-\Delta_{01}^{-}+\frac{N_{01}^{+}-N_{01}^{-}}{N_{01}}\right) \end{array}$$

and hence

$$\begin{array}{ccc} |S_{1}-S_{1}^{\prime }| & ⩽ & \frac{1}{N_{00}-1}\left(|\Delta_{00}^{+}-\Delta_{00}^{-}|+\frac{|N_{00}^{+}-N_{00}^{-}|}{N_{00}}\right)+\frac{1}{N_{01}+1}\left(|\Delta_{01}^{+}-\Delta_{01}^{-}|+\frac{|N_{01}^{+}-N_{01}^{-}|}{N_{01}}\right) \end{array}$$

The change from $x_{42}=00$ to $x_{42}^{\prime }=01$ means that, when we move from the computation of $S_{1}$ to the computation of $S_{1}^{\prime }$, we have one outcome less in regime 00 implying $\Delta_{00}^{+}⩾0$ and $\Delta_{00}^{-}⩾0$. Depending on the table that Alice and Bob used, the change in row 42 may have eliminated a loser or a winner, so there are only two possibilities

$$\begin{array}{c} \left(\Delta_{00}^{+}=0\;\mathrm{and}\;\Delta_{00}^{-}=1\right)\;\mathrm{or}\;\left(\Delta_{00}^{+}=1\;\mathrm{and}\;\Delta_{00}^{-}=0\right), \end{array}$$

which means that $|\Delta_{00}^{+}-\Delta_{00}^{-}|=1$. Similarly, as a result of the change, there is one additional outcome for regime 01, implying $\Delta_{01}^{+}⩽0$ and $\Delta_{01}^{-}⩽0$. The additional outcome maybe a winner or a loser, so similarly, there are only two possibilities

$$\begin{array}{c} \left(\Delta_{01}^{+}=0\;\mathrm{and}\;\Delta_{01}^{-}=-1\right)\;\mathrm{or}\;\left(\Delta_{01}^{+}=-1\;\mathrm{and}\;\Delta_{01}^{-}=0\right), \end{array}$$

which implies $|\Delta_{01}^{+}-\Delta_{01}^{-}|=1$. This simplifies the above estimate to

(26) $$\begin{array}{ccc} |S_{1}-S_{1}^{\prime }| & ⩽ & \frac{1}{N_{00}-1}\left(1+\frac{|N_{00}^{+}-N_{00}^{-}|}{N_{00}}\right)+\frac{1}{N_{01}+1}\left(1+\frac{|N_{01}^{+}-N_{01}^{-}|}{N_{01}}\right)\\ & ⩽ & \frac{2}{N_{00}-1}+\frac{2}{N_{01}}⩽\frac{4}{K}\;, \end{array}$$

because on the set Y, we have $N_{xy}-1⩾K$ for all xy=00,01,10,11.

This line of reasoning is also valid for changes other than the specific change in i=42 from 00 to 01. The fact that the definition of $S_{1}$ has a minus sign in front of the expectation value for regime xy=11 does not pose difficulties. Therefore, we see that $c_{i}=4/K$ provides a general bound in Equation (21) and the proof of Theorem 2 is complete. □

In contrast to Equation (12), Theorems 1 and 2 allow situations where the question regimes do not occur with equal probability. The following Table 3 gives illustrative bounds for these cases. Both Theorems can be useful depending on how the probabilities for the question regimes are set. Theorem 2 can in principle still be improved by using the exact multinomial distribution instead of the estimate made in Equation (23).

## 4. Monte Carlo Simulations and Exploitable Biases

The probability $Pr\{S_{1}^{\mathrm{obs}}⩾2+\eta \}$ can be estimated by performing Monte Carlo simulations on a classical computer. For this, we assume an operationally well-defined strategy that Alice and Bob use to produce their answers a,b and a well-defined mechanism by which the questions x,y are generated. This allows the numerical estimation of bounds without the need for the analytical arguments given in the previous section, but it is of course dependent on specific assumptions regarding the operational procedures.

As discussed in Section 2.1, there are 16 elementary strategies available to Alice and Bob, half of which produce L=3, while the other half produce L=1. If the question regimes are fully generated at random by independent and unbiased coin tosses, i.e., P(xy)=1/4 for all xy=00,01,10,11 in all rounds, and if Alice and Bob just pick randomly from a set of elementary strategies, the probability density for $S_{1}^{\mathrm{obs}}$ will approach the normal distribution with a maximum in the range from −2 to +2. The position of the maximum depends on how often Alice and Bob pick strategies with L=3 versus strategies with L=1.

**Figure 1:**
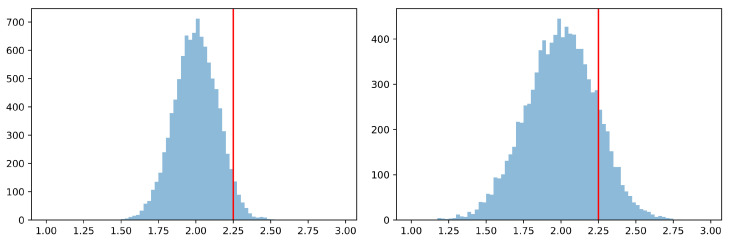
Two probability distributions for $S_{1}^{\mathrm{obs}}$ generated by a Monte Carlo simulation of 10,000 CHSH games of N=500 rounds each. The threshold of 2+η=2.25 is shown in red. (**left**) The graph on the left-hand side was generated with Alice and Bob randomly picking elementary strategies with L=1, while the regimes xy were generated by independent and unbiased coin tosses. The simulated probability is $Pr\{S_{1}^{\mathrm{obs}}⩾2.25\}=4.5\%$ with a maximum value of $S_{1,\max}^{\mathrm{obs}}=2.52$ observed in a single CHSH game. (**right**) The graph on the right-hand side has been generated with Alice and Bob randomly picking elementary strategies that win in the regime xy=00 as well as satisfy L=1. Here, all regimes xy were generated by independent, but biased coin tosses with P(xy=00)=0.7 and P(xy=01)=P(xy=10)=P(xy=11)=0.1. The simulated probability is $Pr\{S_{1}^{\mathrm{obs}}⩾2.25\}=14\%$ with a maximum value of $S_{1,\max}^{\mathrm{obs}}=2.86$ observed in a single CHSH game.

If Alice and Bob want to win and aim for a high number $S_{1}^{\mathrm{obs}}$ while assuming unbiased question regimes, they should use elementary strategies with L=1 only. Assuming that Alice and Bob only pick randomly from those elementary strategies and assuming that the questions are fully generated at random by independent unbiased coin tosses, then a typical probability distribution for $S_{1}^{\mathrm{obs}}$, as shown on the left-hand side of Figure 1, is the result. Here, winning by chance is extremely unlikely if the threshold is set high enough.

If the question regimes are stochastically independent between game rounds but not distributed equally, which may happen in applications of Bell correlations outside physics [20], the chances of winning improve. If, for example, P(xy=00)=:c>0.25 and P(xy=01)=P(xy=10)=P(xy=11)=(1−c)/3 and Alice and Bob are aware of this bias, they should only play elementary strategies with L=1 that are sure to win in regime xy=00. Knowing about such a bias considerably improves the chances of winning, as shown on the right-hand side of Figure 1.

The simulated probability for $S_{1}^{\mathrm{obs}}⩾2+\eta$ remains substantially below the theoretical bounds. This is consistent with what was reported by [27,31], because the bounds based on Hoeffding–Azuma and McDiarmid inequalities are generally not very tight. In addition, some simplifications were made in the proof of Theorem 2, for example, to avoid the use of multinomial distributions.

Clearly, a bias with P(xy=00)=c≫0.25 is easily detectable in the data. However, in case the probability for question regimes varies over the game rounds, things become more tricky. First, note that, for the results in the previous sections, it is not required that the answers from Alice and Bob are stochastically independent over the game rounds. In fact, Alice could simply decide that she will never give the same answer in three consecutive rounds. In this case, *a* is not stochastically independent over the different game rounds, but Theorems 1 and 2 can still be applied. The important assumption only concerns the question regimes.

Systematic biases in random numbers generated from quantum processors do occur in real implementations, so random numbers generated by a quantum computer may not be that random in practice. In [38], various tests were used on random numbers generated by a cloud quantum computer (IBM 20Q Poughkeepsie) and the authors concluded *“As a result, we observed that some qubits were more biased than others”*. Errors and biases can change over time and are difficult to correct, as can be seen in [14], who stated “*However, errors typically fluctuate over time*” and [39] who stated “*While some of the protocols extract quantum randomness and discard deterministic components arisen due to quantum processes implementation imperfections, the fidelity of such procedures is not ideal*”. This is clearly an important consideration when a Bell test is used together with a statistical confidence interval to exclude the possibility of eavesdropping in real-life quantum communication.

We illustrate this problem by a simple scheme, in which the probabilities of the question regimes change predictably between the game rounds. Here, we assume that there is a recurring pattern in the random number generator, such that stochastic patterns repeat every four rounds, as illustrated in Table 4.

This creates an exploitable pattern as the bias rotates over time through the question regimes in a predictable manner. Note that, even in this situation, knowing the question regime drawn in an earlier round still does not give predictive power about the question regimes in later rounds, the predictive power just comes from knowing the round number. As Alice and Bob know, for example, that in round number *n*, where *n* is divisible by 4, the regime xy=00 is more likely to occur, they can just agree to use elementary strategies that maximize the winning probability in such rounds. Thus, it is easy to see that Alice and Bob can exploit this cyclicality by playing appropriate elementary strategies in each game round *n* simply by looking at *n* modulo 4. With a high number of *c*, Alice and Bob thus obtain a good chance of producing values for $S_{1}^{\mathrm{obs}}$ that can be well above 3.

Summing over the columns of Table 4, it is clear that all four question regimes 00,01,10,11 will still occur with an equal probability of around 25% over the entire CHSH game, so the bias only becomes visible when testing the subsets of rounds. Note that Theorems 1 and 2 and related work are not applicable here, because $p_{xy}$ does not stay constant over the game rounds.

## 5. Discussion and Summary

Cheating, such as classical communication between the players of the CHSH game or allowing for an undue influence in the generation of questions, can allow the generation of a quantum-like Bell statistic with classical processes. The amount of cheating that is required is not very large. The Toner–Bacon protocol [40] shows, for example, that when Alice and Bob share two uniformly distributed random variables with values on the unit sphere of $R^{3}$, then sending a single bit can be enough: one bit of information from Alice to Bob suffices to simulate the statistic of a local projective measurement on an entangled Bell pair state. The exchange of one classical bit of information can do a lot in terms of generating strong correlations [41], and in [42], the information to be shared between measurement settings is discussed, illustrating that small amounts of shared classical information suffice in order to break the Bell bound. However, this position [40,41,42] is based on the implicit assumption that an arbitrarily large number of rounds is played and that the question regimes (measurement settings) are produced by unbiased random number generators with equal likelihoods. The practical requirement of having a finite number of game rounds and the possibility of biases were not considered in these contributions. Biases were considered elsewhere [43,44], but with a different focus. The present work addresses statistical questions in a situation where the number of rounds is finite and where biases may be present.

This has practical implications: when CHSH inequalities are used to certify that no eavesdropping took place in quantum communication during a shorter time period, it is natural to question the likelihood of whether any observed violations of CHSH inequalities could have occurred by chance alone. In a pedagogical presentation of entanglement and CHSH games, a similar question may arise: *“How much luck is needed to win a CHSH game without entangled particles?”* The rehearsed opinion that the observed violation could have only occurred with a high standard deviation (“a *n*-sigma violation”) is not a good answer as the a priori assumption of a normal distribution can be dangerous. Good answers do exist in the literature, as can be seen in, e.g., the review in [27], but these tend to be based on highly technical tools and they focus on small biases in the random number generators that provide the question regimes.

In the present work it is assumed that Alice and Bob do not have access to additional resources (like entangled states) while playing the CHSH game. As it is well-known, the availability of such resources (e.g. pairs of entangled qubits for all or some of the rounds of the CHSH game) would give Alice and Bob a distinct advantage and recent work has offered new possibilities for how to represent perfect or imperfect entangled states mathematically [45,46,47]. Note, confidence intervals for the *S*-value that is achievable with such additional resources over a finite number of game rounds may be computed as well. There are interesting corresponding implications, which could be explored in future work.

In the present paper, we focus on an accessible development of bounds for the probability of being lucky in the form of

$$\begin{array}{c} \mathrm{Pr}\{S_{1}⩽2+\eta \}⩾1-q \end{array}$$

for a given threshold, η>0. We discussed different forms of game play and provided three formulae for a straightforward computation of *q*, namely Equations (12), (18) and (24). In contrast to most of the literature, we also covered situations in which the probabilities for different question regimes do not have to be more or less equal. Here, the chances for Alice and Bob to win can improve considerably.

Our extension of the result in [26] is presented with a fully self-contained proof. We did not consider multipartite games, which do not always allow a quantum advantage [48], and where general problems, such as computing the maximum winning probability given an arbitrary amount of entangled particles, can be very complex and even QMA-hard [49].

We analyzed different game strategies in Monte Carlo simulations. We would like to point to the possibility of winning the game by exploiting systematic biases in the generation of question regimes. In such a situation, Alice and Bob can have a good chance of producing $S_{1}$ values substantially above 2 without entangled particles. Therefore, when the security of quantum communication is based on the observed violations of CSHS inequalities, it is important to check the question regimes for systematic biases.

## Figures and Tables

**Table 1:** The following table illustrates the goal implied by Equation (1) for the four possible question regimes and it lists the elementary strategies that result in a loss under each question regime. Instead of showing all four bits of a losing elementary strategy $\left(A_{0},A_{1},B_{0},B_{1}\right)$, only the bits corresponding to the relevant question regime are shown, because the value of the other bits is irrelevant. Note that, in each round, Alice only knows *x* and has to answer by giving the value of *a*, while Bob only knows *y* and has to give the answer *b*.

*x*	*y*	Regime	Needed to Win	Losing Strategies
0	0	xy=00	0=a⊕b	$\left(A_{0},B_{0}\right)=\left(0,1\right)$ or (1,0)
0	1	xy=01	0=a⊕b	$\left(A_{0},B_{1}\right)=\left(0,1\right)$ or (1,0)
1	0	xy=10	0=a⊕b	$\left(A_{1},B_{0}\right)=\left(0,1\right)$ or (1,0)
1	1	xy=11	1=a⊕b	$\left(A_{1},B_{1}\right)=\left(0,0\right)$ or (1,1)

**Table 2:** The following table illustrates the bounds for the certainty that the CHSH game is not won by chance, assuming four equally probable question regimes. Column (a) illustrates the bound obtained with Equation (12), Column (b) illustrates the best available bounds from Theorem 1 and Column (c) illustrates the best available bounds from Theorem 2.

*N*	η	(a)	(b)	(c)
500	0.5	negative	negative	37.73%
1000	0.5	negative	13.51%	69.66%
2500	0.5	30.37%	93.33%	97.23%
5000	0.5	93.94%	99.90%	99.95%

**Table 3:** The following table illustrates the bounds for the certainty that the CHSH game is not won by chance over *N* rounds with threshold η assuming unequal probabilities for the four question regimes. Columns (a) and (c) illustrate the bounds obtained from Theorem 1 and Columns (b) and (d) the bounds from Theorem 2. The values in Columns (a) and (b) were computed for a weakly asymmetric situation with $p_{00}=22\%$ and $p_{01}=p_{10}=p_{11}=26\%$. The values in Columns (c) and (d) were computed for a strongly asymmetric situation with $p_{00}=10\%$ and $p_{01}=p_{10}=p_{11}=30\%$.

*N*	η	(a)	(b)	(c)	(d)
500	0.5	negative	30.91%	negative	4.04%
1000	0.5	12.71%	60.45%	negative	12.35%
2500	0.5	92.91%	93.82%	69.61%	37.20%
5000	0.5	99.87%	99.75%	93.97%	66.35%
500	0.75	14.51%	57.42%	negative	9.36%
1000	0.75	85.39%	87.98%	52.34%	26.32%
2500	0.75	99.92%	99.79%	93.37%	65.46%
5000	0.75	99.99%	99.99%	99.73%	91.58%

**Table 4:** An example for a systematic cyclical bias in the random question regimes that repeats over the round numbers *n*. The number *k* is understood to run through the natural numbers k=1,2,3,….

Round *n*	P(xy=00)	P(xy=01)	P(xy=10)	P(xy=11)
⋯				
4k	*c*	(1−c)/3	(1−c)/3	(1−c)/3
4k+1	(1−c)/3	*c*	(1−c)/3	(1−c)/3
4k+2	(1−c)/3	(1−c)/3	*c*	(1−c)/3
4k+3	(1−c)/3	(1−c)/3	(1−c)/3	*c*
⋯				

## Data Availability

Not applicable.
